# Circulating carotenoids are associated with favorable lipid and fatty acid profiles in an older population at high cardiovascular risk

**DOI:** 10.3389/fnut.2022.967967

**Published:** 2022-09-29

**Authors:** María Marhuenda-Muñoz, Inés Domínguez-López, Klaus Langohr, Anna Tresserra-Rimbau, Miguel Ángel Martínez González, Jordi Salas-Salvadó, Dolores Corella, María Dolores Zomeño, J. Alfredo Martínez, Angel M. Alonso-Gómez, Julia Wärnberg, Jesús Vioque, Dora Romaguera, José López-Miranda, Ramón Estruch, Francisco J. Tinahones, José Lapetra, Ll. Serra-Majem, Aurora Bueno-Cavanillas, Josep A. Tur, Vicente Martín-Sánchez, Xavier Pintó, Miguel Delgado-Rodríguez, Pilar Matía-Martín, Josep Vidal, Clotilde Vázquez, Lidia Daimiel, Emilio Ros, Estefanía Toledo, María Fernández de la Puente Cervera, Rocío Barragán, Montse Fitó, Lucas Tojal-Sierra, Enrique Gómez-Gracia, Juan Manuel Zazo, Marga Morey, Antonio García-Ríos, Rosa Casas, Ana M. Gómez-Pérez, José Manuel Santos-Lozano, Zenaida Vázquez-Ruiz, Alessandro Atzeni, Eva M. Asensio, M. Mar Gili-Riu, Vanessa Bullon, Anai Moreno-Rodriguez, Oscar Lecea, Nancy Babio, Francesca Peñas Lopez, Guadalupe Gómez Melis, Rosa M. Lamuela-Raventós

**Affiliations:** ^1^Centro de Investigación Biomédica en Red Fisiopatología de la Obesidad y la Nutrición, Instituto de Salud Carlos III, Madrid, Spain; ^2^Department of Nutrition, Food Science and Gastronomy, School of Pharmacy and Food Sciences and XIA, Institute of Nutrition and Food Safety, University of Barcelona, Santa Coloma de Gramenet, Spain; ^3^Department of Statistics and Operations Research, Universitat Politècnica de Catalunya-Barcelona TECH, Jordi Girona, Barcelona, Spain; ^4^Department of Preventive Medicine and Public Health, IdiSNA, University of Navarra, Pamplona, Spain; ^5^Departament de Bioquímica i Biotecnologia, Unitat de Nutrició Humana, Universitat Rovira i Virgili, Reus, Spain; ^6^Nutrition Unit, University Hospital of Sant Joan de Reus, Reus, Spain; ^7^Institut d'Investigació Sanitària Pere Virgili, Reus, Spain; ^8^Department of Preventive Medicine, University of Valencia, Valencia, Spain; ^9^Unit of Cardiovascular Risk and Nutrition, Institut Hospital del Mar de Investigaciones Médicas, Barcelona, Spain; ^10^School of Health Sciences, Blanquerna-Ramon Llull University, Barcelona, Spain; ^11^Department of Nutrition, Food Sciences, and Physiology, Center for Nutrition Research, University of Navarra, Pamplona, Spain; ^12^Cardiometabolic Nutrition Group, IMDEA Food, CEI UAM + CSIC, Madrid, Spain; ^13^Bioaraba Health Research Institute, Osakidetza Basque Health Service, Araba University Hospital, University of the Basque Country, Vitoria-Gasteiz, Spain; ^14^Department of Nursing, School of Health Sciences, Instituto de Investigación Biomédica de Málaga, University of Málaga, Málaga, Spain; ^15^CIBER de Epidemiología y Salud Pública, Instituto de Salud Carlos III, Madrid, Spain; ^16^Universidad Miguel Hernandez, Instituto de Investigación Sanitaria y Biomédica de Alicante, Elche-Alicante, Spain; ^17^Health Research Institute of the Balearic Islands (IdISBa), Palma de Mallorca, Spain; ^18^Department of Internal Medicine, Maimonides Biomedical Research Institute of Cordoba, Reina Sofia University Hospital, University of Cordoba, Cordoba, Spain; ^19^Department of Internal Medicine, Hospital Clínic, Institut d'Investigacions Biomèdiques August Pi Sunyer, University of Barcelona, Barcelona, Spain; ^20^Department of Endocrinology, Virgen de la Victoria Hospital, Instituto de Investigación Biomédica de Málaga, University of Málaga, Málaga, Spain; ^21^Research Unit, Department of Family Medicine, Distrito Sanitario Atención Primaria Sevilla, Sevilla, Spain; ^22^Research Institute of Biomedical and Health Sciences, University of Las Palmas de Gran Canaria, Las Palmas de Gran Canaria, Spain; ^23^Centro Hospitalario Universitario Insular Materno Infantil, Canarian Health Service, Las Palmas de Gran Canaria, Spain; ^24^Department of Preventive Medicine and Public Health, University of Granada, Granada, Spain; ^25^Research Group on Community Nutrition and Oxidative Stress, IUNICS, University of Balearic Islands, Palma de Mallorca, Spain; ^26^Institute of Biomedicine, University of León, León, Spain; ^27^Lipids and Vascular Risk Unit, Internal Medicine, Hospital Universitario de Bellvitge, Hospitalet de Llobregat, Barcelona, Spain; ^28^Division of Preventive Medicine, Faculty of Medicine, University of Jaén, Jaén, Spain; ^29^Department of Endocrinology and Nutrition, Instituto de Investigación Sanitaria Hospital Clínico San Carlos, Madrid, Spain; ^30^CIBER Diabetes y Enfermedades Metabólicas, Instituto de Salud Carlos III, Madrid, Spain; ^31^Department of Endocrinology, Hospital Clínic, IDIBAPS, University of Barcelona, Barcelona, Spain; ^32^Department of Endocrinology and Nutrition, Hospital Fundación Jimenez Díaz, Instituto de Investigaciones Biomédicas, University Autonoma, Madrid, Spain; ^33^Nutritional Control of the Epigenome Group, Precision Nutrition and Obesity Program, IMDEA Food, CEI UAM + CSIC, Madrid, Spain; ^34^Centro de Salud Raval, Elche-Alicante, Spain; ^35^Department of Preventive Medicine and Public Health, School of Medicine, Instituto de Investigación Biomédica de Málaga, University of Málaga, Málaga, Spain; ^36^Department of Family Medicine, Atención Primaria Servicio Navarro de Salud, Pamplona, Spain

**Keywords:** Mediterranean diet, PREDIMED-plus study, plasma carotenoids, cardiovascular health, liquid chromatography, mass spectrometry

## Abstract

Carotenoid intake has been reported to be associated with improved cardiovascular health, but there is little information on actual plasma concentrations of these compounds as biomarkers of cardiometabolic risk. The objective was to investigate the association between circulating plasma carotenoids and different cardiometabolic risk factors and the plasma fatty acid profile. This is a cross-sectional evaluation of baseline data conducted in a subcohort (106 women and 124 men) of an ongoing multi-factorial lifestyle trial for primary cardiovascular prevention. Plasma concentrations of carotenoids were quantified by liquid chromatography coupled to mass spectrometry. The associations between carotenoid concentrations and cardiometabolic risk factors were assessed using regression models adapted for interval-censored variables. Carotenoid concentrations were cross-sectionally inversely associated with serum triglyceride concentrations [−2.79 mg/dl (95% CI: −4.25, −1.34) and −5.15 mg/dl (95% CI: −7.38, −2.93), *p*-values = 0.0002 and <0.00001 in women and men, respectively], lower levels of plasma saturated fatty acids [−0.09% (95% CI: −0.14, −0.03) and −0.15 % (95% CI: −0.23, −0.08), *p*-values = 0.001 and 0.0001 in women and men, respectively], and higher levels of plasma polyunsaturated fatty acids [(0.12 % (95% CI: −0.01, 0.25) and 0.39 % (95% CI: 0.19, 0.59), *p*-values = 0.065 and 0.0001 in women and men, respectively] in the whole population. Plasma carotenoid concentrations were also associated with higher plasma HDL-cholesterol in women [0.47 mg/dl (95% CI: 0.23, 0.72), *p*-value: 0.0002], and lower fasting plasma glucose in men [−1.35 mg/dl (95% CI: −2.12, −0.59), *p*-value: 0.001].

## Introduction

The effect of Mediterranean diet on reducing the incidence of cardiovascular disease and mortality has been established with first level evidence by the researchers working on randomized trials and high-quality prospective cohort studies like the PREDIMED (PREvención con DIeta MEDiterránea) randomized trial (1–5) and others (6–10). The success of this study led to the design of the PREDIMED-Plus study, which implemented a healthy Mediterranean lifestyle adding physical activity and behavioral changes to an energy-restricted Mediterranean diet in order to evaluate the effect of weight loss on cardiovascular disease (11). Both interventions considered fruits and vegetables to be essential in preventive cardiology due to their content in fiber and phytochemicals. In fact, the intervention in the PREDIMED-Plus trial targeted fruits and vegetables and demonstrated a substantial increment in their consumption after 6 and 12 months follow-up (12).

Carotenoids are one of the groups of phytochemicals present in these foods, being natural pigments that give them their yellow to reddish tonalities (13). Some, but not all of them, can be converted into vitamin A by the human body, known for its role in controlling gene transcription (14).

In recent years, several studies and systematic reviews have focused on the health effects of carotenoids. Possibly due to their antioxidant capacity, consumption of carotenoid-rich foods has been associated with the prevention and control of type 2 diabetes and some of its complications (15) and they are also believed to have a role in the risk reduction of developing cardiovascular diseases (16, 17) and metabolic syndrome, which is characterized by impaired glucose metabolism and abnormal lipid profile, together with elevated blood pressure and abdominal obesity (18, 19).

This study aims to assess the association of plasma carotenoid concentrations in an older population with metabolic syndrome with anthropometric, clinical, and biochemical parameters related to cardiovascular risk.

## Materials and methods

### Study design

This work was performed as a cross-sectional analysis of baseline data of a subgroup of participants in the PREDIMED-Plus study (20), for which eligible subjects met at least three of the metabolic syndrome criteria described by the International Diabetes Federation, the American Heart Association, and the National Heart, Lung, and Blood Institute (21). Participants were women and men between 55 and 75 years and overweight or obese (body mass index between 27 and 40 kg/m^2^). The selection criteria and description of the PREDIMED-Plus cohort have been detailed elsewhere (11). The complete study protocol can be found at http://www.predimedplus.com/ (accessed on 16 May 2021).

For the present study K3-EDTA plasma samples from the baseline were selected according to the evaluation given by the baseline semi-quantitative 143-item PREDIMED-Plus food frequency questionnaires (22). The total PREDIMED-Plus study population was divided into fruit and vegetable consumption deciles and fat intake quartiles. Only participants belonging to the extreme fruit and vegetable consumption (deciles 1 and 10) and the extreme fat intake (quartiles 1 and 4) were included in the study (*n* = 230). Even though the food intake was clearly compartmentalized, the plasma concentrations of carotenoids were not, as we previously described (23), they were dispersed ranging from 0 to more than 40 μmol/L.

### Ethics statement

The PREDIMED-Plus study was carried out in accordance with The Code of Ethics of the World Medical Association (Declaration of Helsinki) for experiments involving humans, and all procedures involving human participants were approved by the Institutional Review Boards of the participating centers. The clinical trial was registered in the ISRCTN of London, England with the number 89898870 on 24 July 2014. Written informed consent was obtained from all participants.

### Outcome variables

#### Anthropometric and body composition variables

Anthropometric measurements were performed by trained personnel according to the PREDIMED-Plus protocol. Weight was recorded using calibrated scales with barefoot participants wearing light clothing. Height was measured with wall-mounted stadiometers. Body mass index was calculated as weight (kg) divided by the squared height (m^2^). Waist circumference was measured using non-stretchable measuring tapes midway between the lowest rib and the iliac crest with subjects standing at the end of gentle expiration.

#### Blood pressure

Systolic and diastolic blood pressure and heart rate were measured in triplicate with semiautomatic oscillometers (Omron HEM-705CP).

#### Biochemical parameters

Determination of glucose (mg/dl), glycated hemoglobin (%), triglycerides (mg/dl), total cholesterol, and HDL-cholesterol (mg/dl) was performed by standard laboratory enzymatic methods in the clinical analysis cores of each PREDIMED-Plus participating center.

#### Plasma fatty acid profile

##### Samples-standards-and-reagents

Baseline K3-EDTA fasting plasma samples obtained in the first study visit were analyzed. These samples had been stored at – 80°C until use. Tridecanoic acid methyl ester (C13:0), boron trifluoride-methanol reagent, n-hexane, and sodium methylate (0.5% w/v) were acquired from Sigma-Aldrich (St. Louis, MO, USA). Sodium chloride solution was purchased from Panreac Quimica SLU (Barcelona, Spain) and anhydrous sodium sulfate from Schalab (Barcelona, Spain). Supelco 37 component fatty acids methyl ester mix and PUFA No.2 (Animal source) were acquired from Merck (Darmstadt, Germany).

##### Fatty-acid-derivatization

The determination of fatty acids was performed using a validated method (24). Briefly, 20 μl of the internal standard tridecanoic acid methyl ester (C13:0) were added to 100 μl of plasma samples. Then, 1 ml of sodium methylate (0.5% w/v) was added, and the mix was heated to 100°C for 15 min. After cooling, samples were esterified with 1 ml of boron trifluoride-methanol reagent, again at 100°C, for 15 min. When the sample was cool, fatty acids methyl esters were isolated by adding 500μl of n-hexane. The mix was vortexed for 1 min, then, 1 ml of a saturated sodium chloride solution was added, and the tubes were centrifuged for 10 min at 3,000 rpm at room temperature. After drying with anhydrous sodium sulfate, the clear n-hexane top layer was transferred into an automatic injector vial equipped with a volume adapter of 300 μl.

##### Gas-chromatography-conditions

Fast gas chromatography analyses were performed on a Shimadzu GC-2010 Gas Chromatograph (Shimadzu, Kyoto, Japan) equipped with a flame ionization detector and a Shimadzu AOC-20i Autoinjector. Separation of fatty acids methyl esters was carried out on a capillary column (40 m × 0.18 mm I.D. × 0.1 μm film thickness) coated with an RTX-2330 stationary phase of 10% cyanopropyl phenyl −90% byscyanopropyl polysiloxane from Restek (Bellefonte, USA).

Operating conditions were as follows: the split-splitness injector was used in split mode with a split ratio of 1:50. The injection volume of the sample was 1 μl. The injector and detector temperatures were kept at 250 and 300°C, respectively. The program initial temperature was 110°C, increased at 52°C/min to 195°C and held at this temperature for 6 min, then it was increased at 25°C/min to 230°C and held for 6.5 min (total run time: 16.03 min). Hydrogen was used as the carrier gas at a constant pressure of 26 psi that refers to a linear velocity of 40 cm/s at 110°C. Data acquisition and processing were performed with the Shimadzu-Chemstation software for GC systems. Supelco 37 component fatty acids methyl ester mix and PUFA No.2 (Animal source) were used for peak identification. Results were expressed as relative percentages of total FA.

### Exposure variables

The total sum of carotenoids in plasma and of the two groups of carotenoids: carotenes (α-carotene, β-carotene, *E*-lycopene, and *Z*-lycopene) and xanthophylls (astaxanthin, lutein, canthaxanthin, and β-cryptoxanthin) were computed from the concentration of the individual carotenoids calculated after liquid-liquid extraction and separation by liquid chromatography coupled to UV-VIS detector and a triple quadrupole mass spectrometer, following the procedure described by Hrvolová et al. (25). Due to the limits of detection and quantification of the method, the concentrations were interval-censored.

#### Samples, standards, and solvents

The analyzed samples were −80°C stored K3-EDTA fasting plasma that had been drawn at baseline. Handling of all samples and standards was always done under cool conditions and avoiding exposure to light. Carotenoid standards: astaxanthin, canthaxanthin, *E*-β-apo-8′-carotenal, α-carotene, β-carotene, fucoxanthin, and lycopene were obtained from Sigma-Aldrich (St. Louis, MO, USA). Lutein was purchased from Cayman Chemical (Ann Arbor, MI, USA), zeaxanthin and β-cryptoxanthin were provided by Extrasynthese (Genay, Lyon, France). 13-*Z*-β-carotene and 9-*Z*-β-carotene were purchased from Carbosynth (Newbury, Berkshire, UK).

Methanol of LC-MS grade, n-hexane, ethanol, and methyl tert-butyl ether (MTBE) of HPLC grade, blank human plasma, and butylated hydroxytoluene (BHT) were obtained from Sigma-Aldrich. Ammonium acetate (AMAC) and acetic acid of HPLC grade were purchased from Panreac Quimica SLU (Barcelona, Spain). Ultrapure water (Milli-Q) was generated by a Millipore system (Bedford, MA, USA).

#### Carotenoid extraction and analysis

The extraction of carotenoids was performed using a previously validated method with minor modifications (26). Briefly, 450 μl of plasma were thawed and mixed with 800 μl of ethanol, 500 μl of ultrapure water, and 2 ml of n-hexane/BHT (100 mg/L). Hundred microliters of fucoxanthin at 1 mg/ml were also added as internal standard. After vortexing for 1 min and centrifugating at 2,070 × *g* for 5 min at 4°C and the upper non-polar layer was separated into a new tube and the lower aqueous phase underwent re-extraction with two more milliliters of n-hexane/BHT (100 mg/L). Both upper non-polar layers were combined and evaporated to dryness under nitrogen stream at room temperature. The evaporate was reconstituted with 100 μl of methanol and stored in glass amber vials at −80°C until analysis.

Calibration curves were prepared following the same procedure using stock solution of blank human plasma and carotenoid standards.

Quantitation of carotenoids was achieved by means of separation by liquid chromatography with a YMC Carotenoid S-5 μm, 250 × 4.6 mm (Waters, Milford, MA, USA) and detection with UV-VIS detector set at 450 nm and mass spectrometer triple quadrupole mass spectrometer equipped with APCI ionization source using a published method by our group (25) with minor modifications, namely, the implementation of internal standard method for quantitation (23). MultiQuant software version 3.0.1 (Sciex) was used for chromatographic analysis. Due to the labile profile of the *Z*-lycopene standard, this carotenoid was quantified in *E*-lycopene equivalents.

### Covariates and other variables

Information related to sociodemographic and lifestyle habits, individual and family medical history, medical conditions, and medication use were evaluated using self-reported questionnaires with the support of trained personnel (11). From the validated food frequency questionnaire, we also estimated the total energy intake in kcal/day. Finally, the quality of the diet was assessed based on the validated 17-item energy-reduced Mediterranean diet adherence score.

### Statistical analyses

Descriptive characteristics are presented using means and standard deviations for continuous variables and percentages for categorical data. Linear regression models were used to examine the cross-sectional association between plasma carotenoids and anthropometric, clinical, and biochemical parameters of cardiovascular risk. The carotenoid's regression coefficient represents the effect size measure of these models and corresponds to the expected change in the dependent variable per unit increase of plasma carotenoids (μmol/L). The method applied to fit these models considered values of plasma carotenoids below the detection and quantification, which are, actually, interval-censored data (i.e., they are not observed exactly and as a consequence, they lie within an interval) (27, 28). The use of carotenoids as a continuous variable allows the analysis to be done without losing information. On the other hand, due to the interval-censoring of the data it is not possible to make quantiles without making imputation, since the intervals overlap.

The analyses were also stratified by sex according to the recommendations of the Department of Economic and Social Affairs of the United Nations Secretariat (29). All analyses were adjusted for age, body mass index, leisure time physical activity, total energy intake, and adherence to the Mediterranean diet, all continuous variables, unless the variable was being tested. Blood pressure was also adjusted for anti-hypertensive agents and triglycerides for cholesterol-lowering agents. The model assumptions of homoscedasticity and normality were graphically checked by means of residual plots.

Analyses were performed with the statistical software R, version 4.1.0 (Vienna, Austria; https://www.r-project.org/). Statistical significance was set at <0.05.

## Results

The general characteristics of the studied population are summarized in Table 1. Overall, men were slightly younger, more active, and had higher total energy intake than women. Their intake of alcohol and percentage of caloric intake as protein were also higher.

**Table 1:** General characteristics of the population.

**Characteristics**	**All**	**Women**	**Men**
No. of subjects	230	106	124
Age, years	66.1 ± 4.40	66.8 (4.43)	65.5 (4.24)
Type 2 diabetes, *n* (%)	55 (23.6)	21 (19.8)	34 (27.4)
Hypercholesterolemia, *n* (%)	155 (67.4)	78 (73.6)	77 (62.1)
Hypertension, *n* (%)	200 (87.0)	90 (84.9)	110 (88.7)
Medications, *n* (%)			
Insulin	8 (3.5)	1 (0.9)	7 (5.7)
Metformin	44 (19.1)	16 (15.1)	28 (22.6)
Other hypoglycemic drugs	37 (16.1)	13 (12.3)	24 (19.4)
Cholesterol-lowering agents	115 (50.0)	54 (50.9)	61 (49.2)
Anti-hypertensive agents	191 (83.0)	86 (81.1)	105 (84.7)
Leisure time physical activity, MET·min/week	2,525 ± 2,458	1,995 (1829)	2,979 (2819)
Body mass index, kg/m^2^	32.7 ± 3.50	33.0 (3.77)	32.3 (3.27)
Current smoker, *n* (%)	37 (16.1)	12 (11.3)	25 (20.2)
Dietary intake			
Total energy intake, kcal/day	2,400 ± 716	2,268 ± 705	2,513 ± 708
Carbohydrate, % kcal/day	41.8 ± 8.19	42.0 ± 8.16	41.6 ± 8.25
Protein, % kcal/day	16.4 ± 3.03	17.2 ± 2.90	15.7 ± 2.97
Fat, % kcal/day	38.1 ± 8.06	39.1 ± 8.16	37.4 ± 7.92
Alcohol, g/day	12.8 ± 16.92	5.42 ± 9.33	19.2 ± 19.3
Fruit and vegetable, g/day	783 ± 544	816 ± 557	755 ± 533
Mediterranean diet adherence score (range 0–17)	8.4 ± 2.9	8.7 ± 2.8	8.2 ± 3.0

MET, metabolic task equivalents. Values are number and percentages for categorical variables and means ± SD for continuous variables.

The association between plasma carotenoids and outcome variables has been performed taking into account the interval-censoring of the quantitated carotenoids.

### Anthropometric and clinical variables

**Figure 1:**
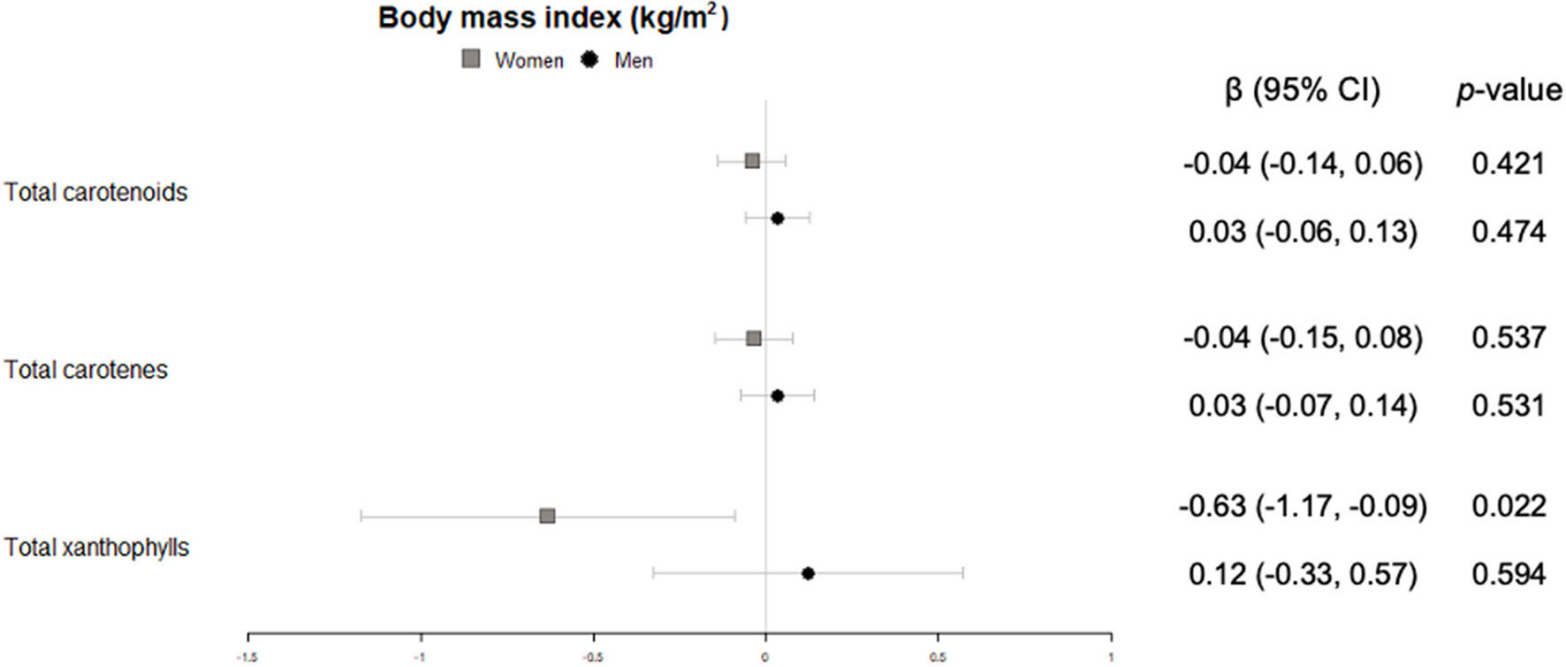
Cross-sectional association of plasma carotenoids and subclasses (per unit increase) with body mass index (kg/m^2^) (95%-CI), and their respective *p*-values.

Lower body mass index in women was significantly associated with higher concentrations of xanthophylls in plasma (Figure 1).

**Figure 2:**
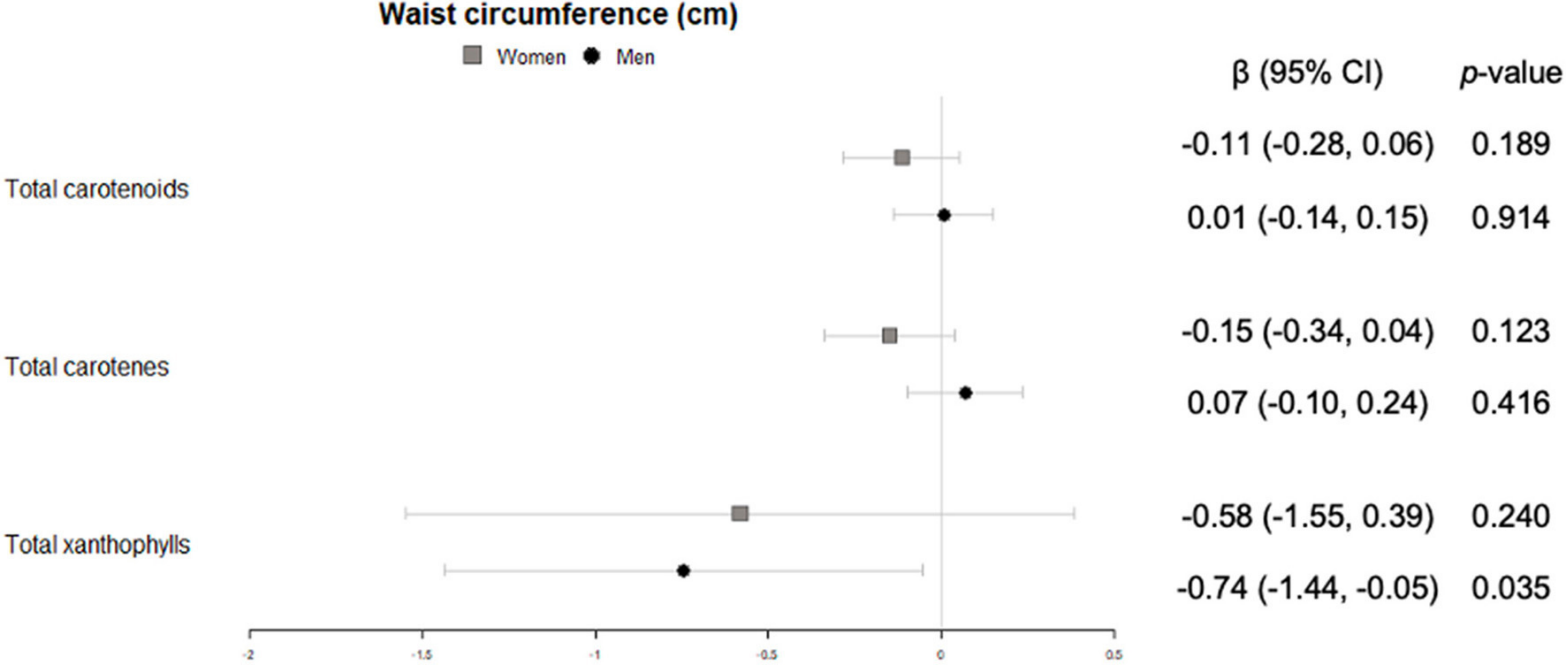
Cross-sectional association of plasma carotenoids and subclasses (per unit increase) with waist circumference (cm) (95%-CI), and their respective *p*-values.

**Figure 3:**
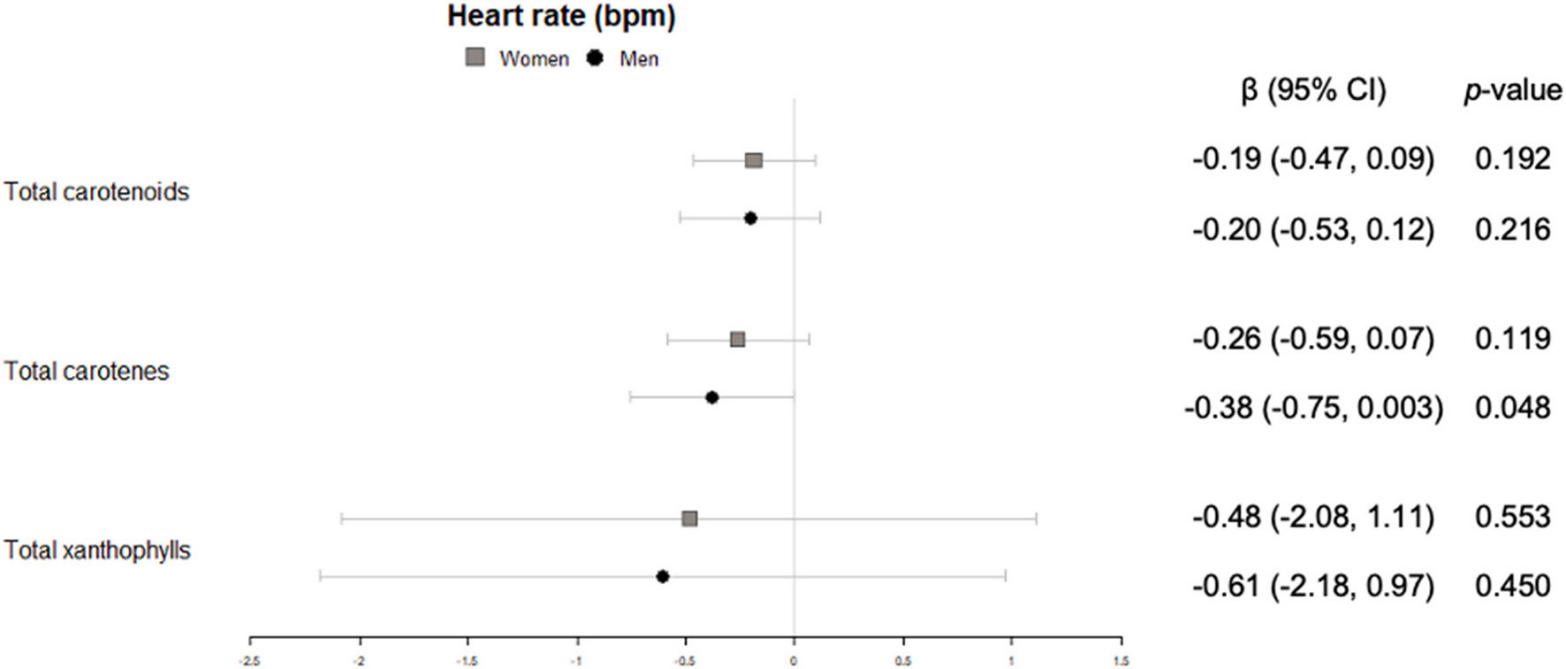
Cross-sectional association of plasma carotenoids and subclasses (per unit increase) with heart rate (bpm) (95%-CI), and their respective *p*-values.

**Figure 4:**
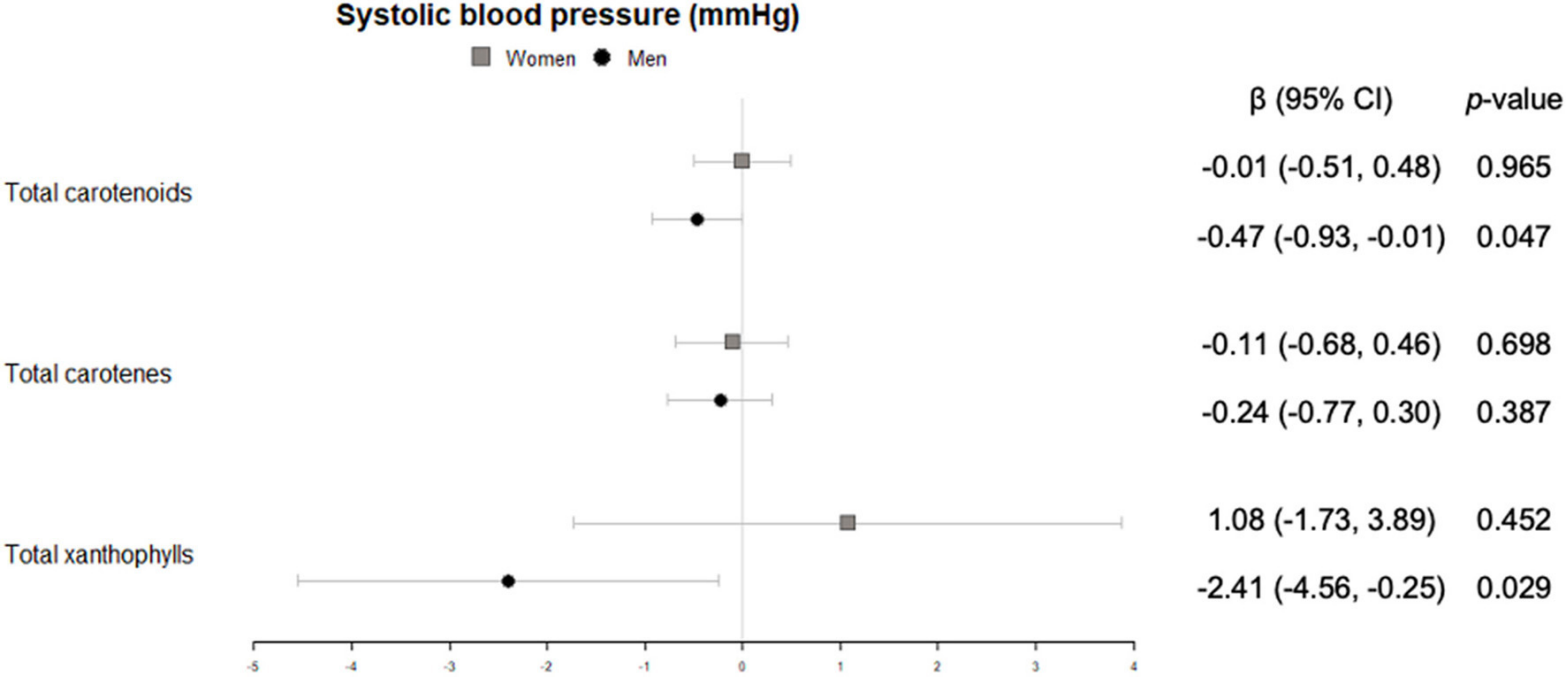
Cross-sectional association of plasma carotenoids and subclasses (per unit increase) with systolic blood pressure (mmHg) (95%-CI), and their respective *p*-values.

In men, significant inverse associations were observed for xanthophyll plasma concentrations with waist circumference (Figure 2), total carotenes with heart rate (Figure 3), and total carotenoids, as well as xanthophylls, with systolic blood pressure (Figure 4).

### Biochemical parameters of cardiovascular health and risk

#### Glucose metabolism

**Figure 5:**
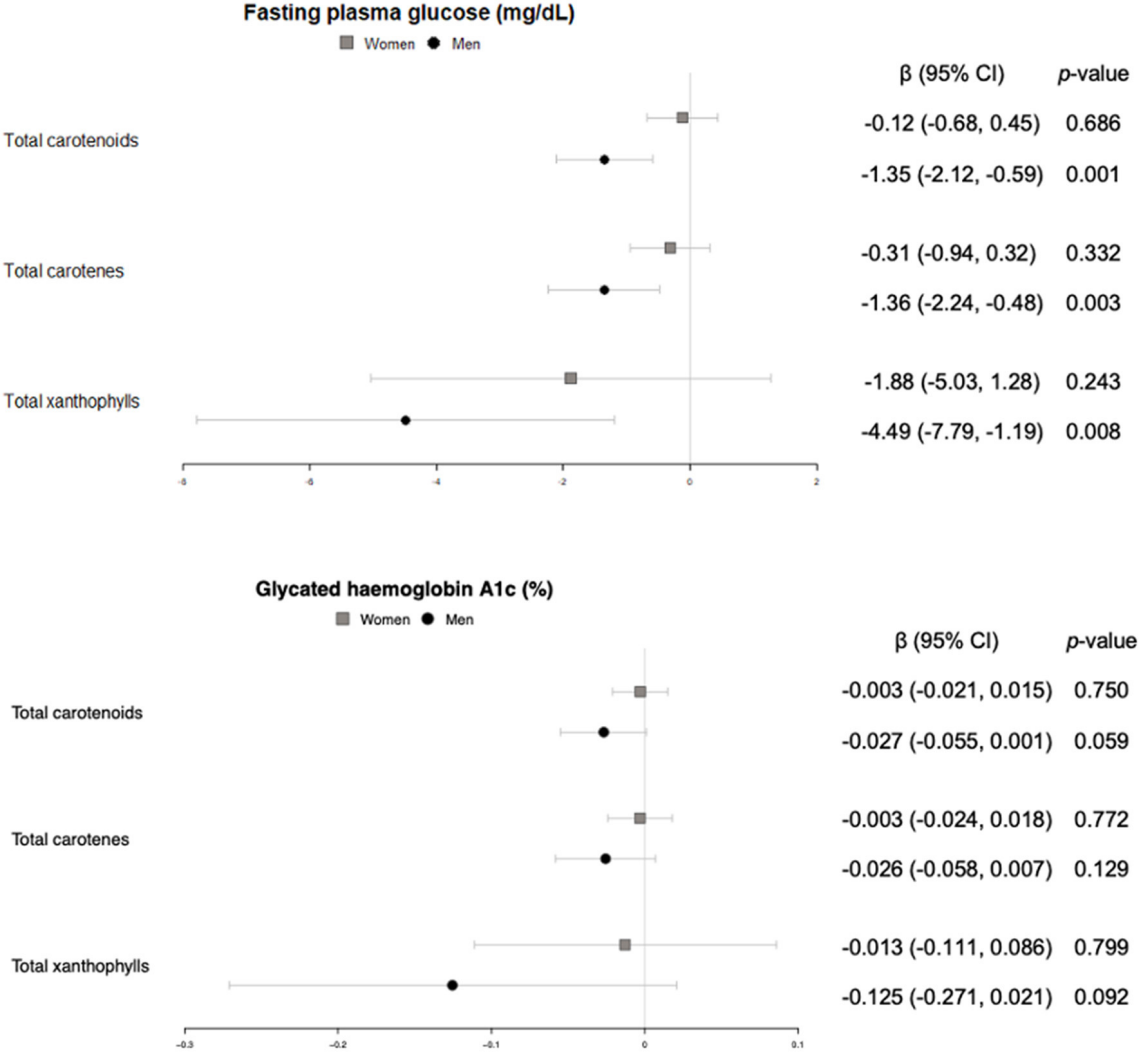
Cross-sectional association of plasma carotenoids and subclasses (per unit increase) with fasting plasma glucose (mg/dl) and glycated hemoglobin A1c (%) (95%-CI), and their respective *p*-values.

Men with higher carotenoid concentrations, both carotenes and xanthophylls, showed significantly lower levels of plasmatic glucose (Figure 5). Additionally, a trend toward lower levels of glycated hemoglobin A1c was observed for higher carotenoid and xanthophyll concentrations (Figure 5). These associations were not found in women.

#### Lipid profile

**Figure 6:**
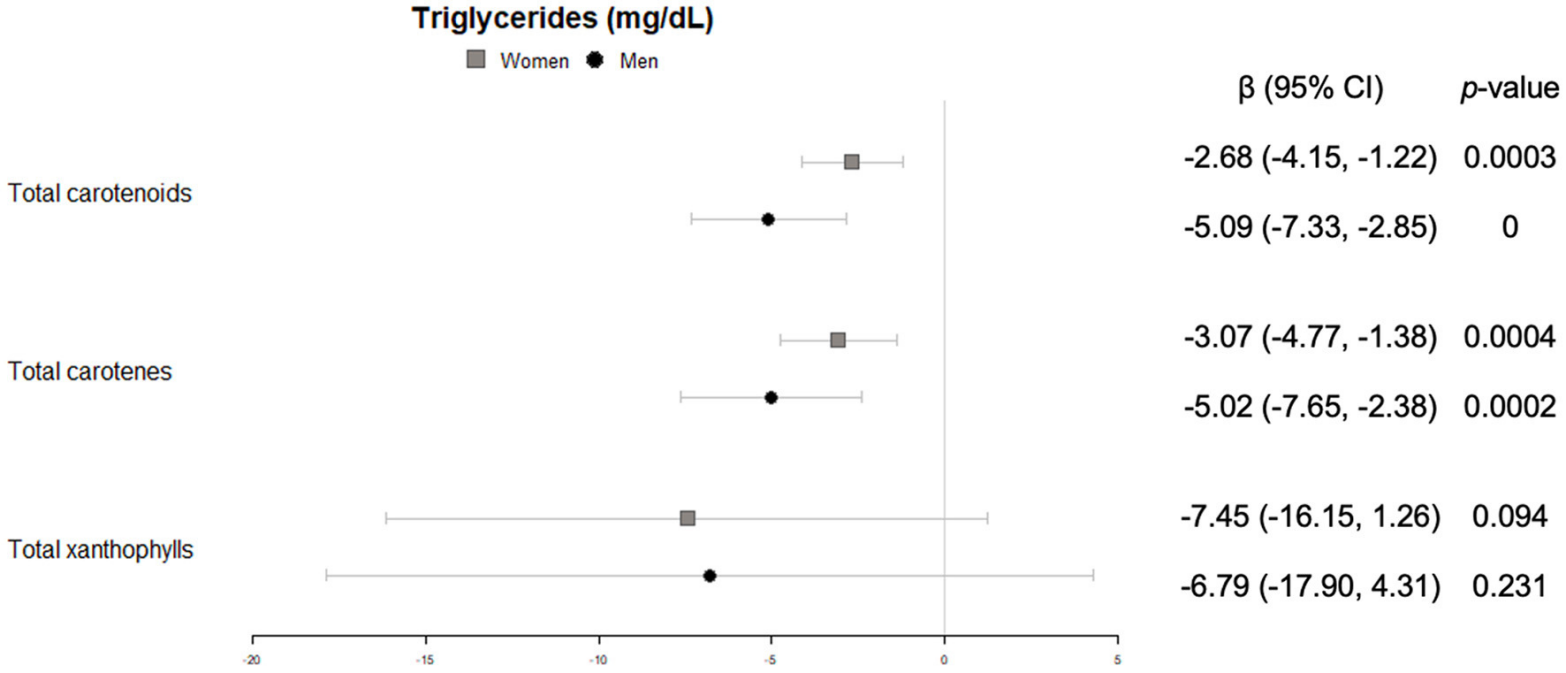
Cross-sectional association of plasma carotenoids and subclasses (per unit increase) with blood triglycerides (mg/dl) (95%-CI), and their respective *p*-values.

Triglycerides were significantly and inversely associated with concentrations of total carotenoids and carotenes in plasma, in both women and men, whereas only in women a trend was found for the sum of xanthophylls (Figure 6). No significant association neither trend was found for total cholesterol in our study.

**Figure 7:**
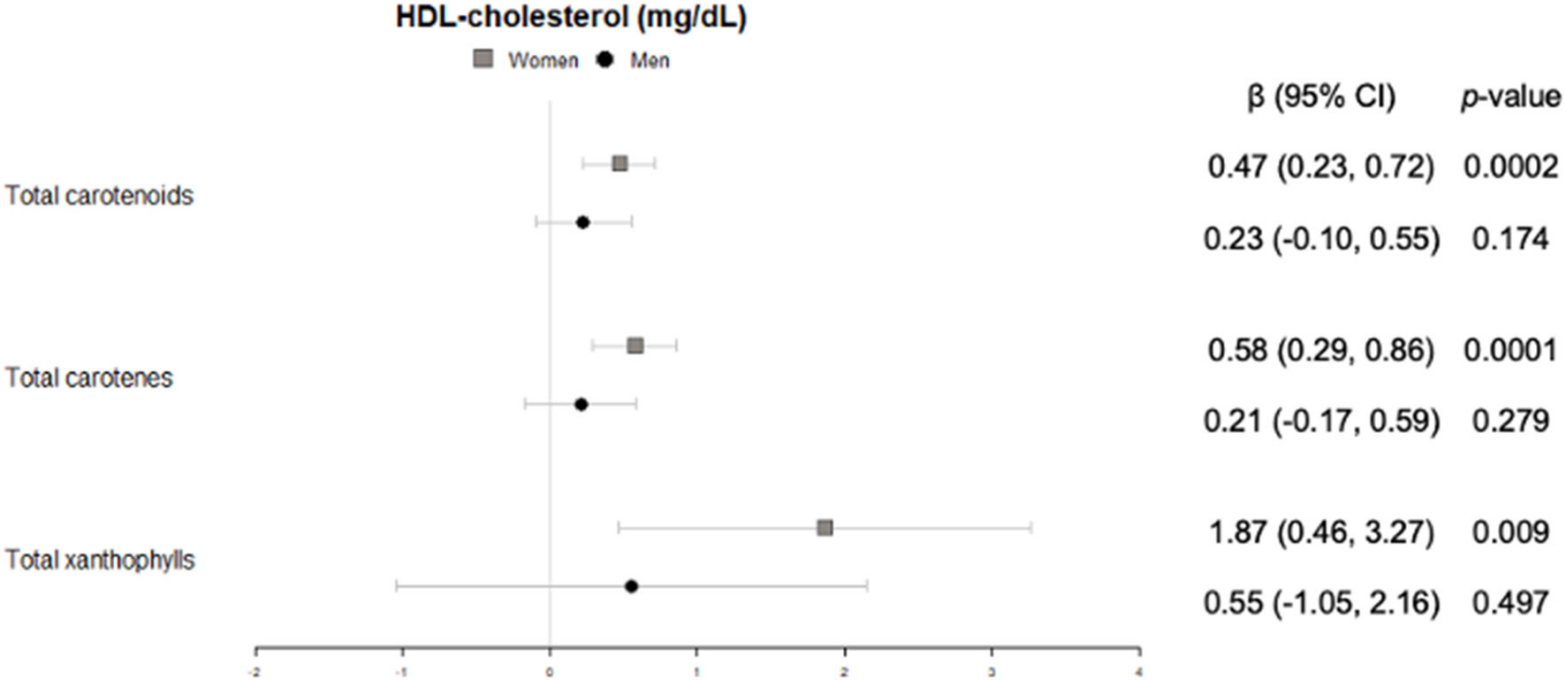
Cross-sectional association of plasma carotenoids and subclasses (per unit increase) with blood HDL-cholesterol (mg/dl) (95%-CI), and their respective *p*-values.

Likewise, only in women HDL-cholesterol showed a significantly positive correlation with total carotenoids, together with both carotenes and xanthophylls (Figure 7).

#### Fatty acid profile

**Figure 8:**
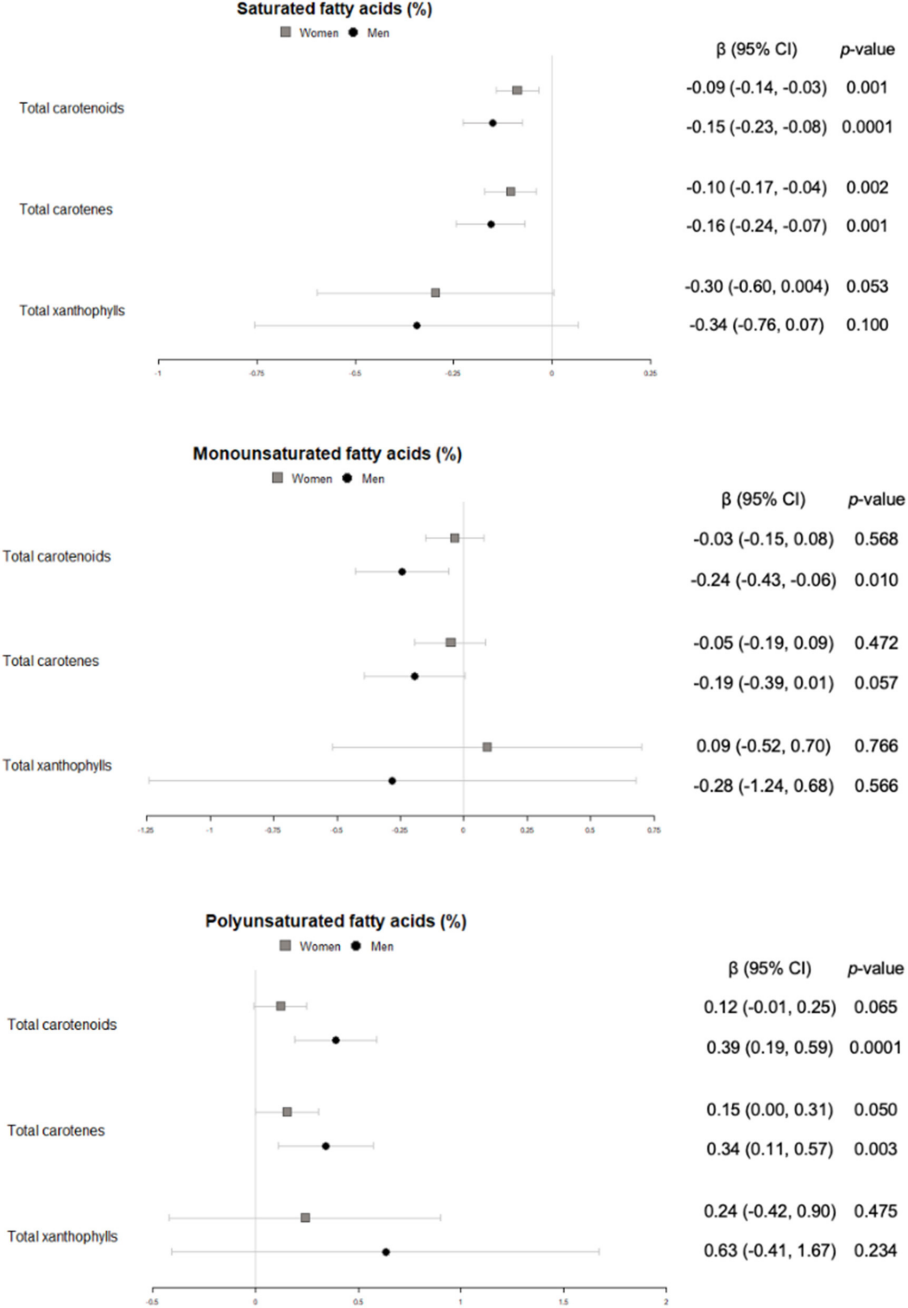
Cross-sectional association of plasma carotenoids and subclasses (per unit increase) with plasma saturated, monounsaturated and polyunsaturated fatty acids (%) (95%-CI), and their respective *p*-values.

Total carotenoids and the sum of carotenes were inversely correlated with saturated fatty acids in all individuals (men and women), and a trend toward the same direction was found for the sum of xanthophylls in women (Figure 8). Regarding monounsaturated fatty acids, no association was observed in women but a significant inverse association for total carotenoids, and a trend in the same direction for carotenes, were found in men (Figure 8). Finally, we uncovered a trend toward association between polyunsaturated fatty acids and total carotenoids in women. This association was significant for men and, when looking at carotenes, in all individuals (Figure 8).

Regarding the possible differences in provitamin A activity we also performed the analyses classifying into provitamin A and non-provitamin A carotenoids, however, no associations neither trends in any of the groups were found.

## Discussion

In this article, we summarize the cross-sectional associations between plasma carotenoid concentrations and anthropometric, clinical, and biochemical parameters in a senior population with overweight/obesity and metabolic syndrome. High plasma concentrations of carotenoids have been found to be associated with a favorable lipid profile. In addition, carotenoid concentrations in men, but not in women, were associated with lower levels of plasma glucose.

Carotenoids have been studied for their antioxidant properties and individual supplementation of carotenoids or carotenoid rich foods have been used in order to assess their metabolic and health attributes. In addition, the provitamin A fraction of carotenoids has also been attributed a role in obesity and cardiovascular disease (30). Higher plasma concentrations of these molecules have been correlated with lower overall and cause-specific mortality in male smokers (31). However, most studies have only considered carotenoid intake, not plasma concentrations, which might not perfectly correlate. In addition, although sex differences have been studied regarding carotenoid levels, they have been less sought regarding cardiovascular risk factors within the same population. In this study, the sample was stratified by sex in order to adress these differences, a practice that is well-recommended by the Statistical Division of the United Nations (29).

Antropometric variables such as body mass index and waist circumference have traditionally been primary attributes of non-communicable preventive diseases. Meta-analyses have reported that carotenoid intake was associated with reduced waist circumference in people with type 2 diabetes, metabolic syndrome, coronary artery disease (32), or individuals with overweight/obesity (33). Our results show that total xanthophylls in men are indeed associated with a lower waist circumference. This concurs with results of a recent study examining sex differences in circulating carotenoids (34). In that study, body mass index also correlated with carotenoid plasma concentrations, a finding that we only observed for xanthophylls in women.

Regarding heart rate and blood pressure, which are non-invasive clinical markers of cardiovascular health, few studies have associated these variables with intake or plasma concentration of carotenoids. In our study, beneficial associations of high carotenoid plasma concentration with and heart rate and systolic blood pressure were observed only for men. A metaanalysis of lycopene interventions (35) and an 8-week randomized controlled trial with astaxanthin supplementation to participants with type 2 diabetes (36) described an association between these carotenoids and systolic blood pressure in the same direction that in our study.

Even though there is abundant information on differences in glucose metabolism between men and women, the underlying causes of which are not yet understood, the literature on sex-related differences in glucose metabolism in relation to carotenoids is scarce because most studies do not stratify participants by sex and the not out of the question differences remain diluted in the overall population (37). In the Botnia Dietary Study, opposite associations between β-carotene and fasting plasma glucose by sex were reported: inverse for dietary intake in men, and direct for plasma concentrations in women (38); while data from the Women's Health Study showed no association between plasma lycopene concentrations and the risk of type 2 diabetes incidence in middle-aged and older women (39). Similarly, we found a significant inverse relationship between glucose and plasma carotenoids, and a trend toward the same outcome for glycated hemoglobin A1c in men, but no association in women. Another study that also stratified their population by sex did find an inverse association for women, but not for overweight individuals (40), which is in line with the lack of association observed in our study, where all individuals were overweight and most of them were obese.

With regard to the lipid profile, there is a general understanding that carotenoids are beneficially associated with lower plasma triglycerides and higher HDL-cholesterol (41), two recognized risk markers of cardiovascular disease. Indeed, we observed that the higher the carotenoid concentration in plasma, the lower the triglyceride levels, both in women and men. This, together with the lack of association with serum total cholesterol concentrations and a direct association with HDL-cholesterol in women, adds to the evidence that carotenoids could play a role in maintaining a salutary lipid profile, even in an older population at high cardiovascular risk. These results could be explained by the enhancement of long-chain fatty acids hydrolisis and mitochondrial and peroxisomal β-oxidation related enzimes induced by the carotenoids (42, 43).

The association between plasma fatty acids and carotenoids has not been thoroughly studied in large cohorts. Not only there is still room for research in carotenoid absorption—regarding micelle formation and intestinal transport, among other variables—depending on the type and amount of fat that is ingested, but also for how they interact once they circulate in blood. In this environment, the lipophilic nature of the carotenoids might prompt a beneficial synergy when partitioning into the chylomicrons. The healthy Mediterranean diet is both rich in carotenoids and characterized by a lower full-fat dairy produce and processed meat consumption, which could also affect the fatty acid profile. In the present study, we observed that plasma carotenoids were directely associated with polyunsaturated fatty acids and inversely associated with saturated fatty acids in the whole sample, while in men an inverse relationship with monounsaturated fatty acids was also found. Similar to our results, in a study with females from a breast cancer trial the investigators detected an increase on carotenoid intake that was not significant when assessing plasma concentrations, but that was concomitant with a decrease in short chain fatty acids, an increase in polyunsaturated fatty acids and a favorable decrease in the ratio of n-6 to n-3 polyunsaturated fatty acids (44). On the contrary, another study with beta carotene, lutein and lycopene supplementation to healthy non-smoker males, researchers found no associations with lutein, a positive association of beta carotene with linoleic acid and a negative one of lycopene with this same polyunsaturated fatty acid (45). More research is needed to uncover these contradictions and focused on the molecular mechanisms that would lead to the association toward a favorable fatty acid profile when blood carotenoids increase.

Our study has limitations due to a small sample size and the cross-sectional design. Coming from a large cohort, the experimental analyses and stratification by sex reduced the number of observations by group. In addition, the scarce literature on sex-related differences in the association of carotenoids (both intake and plasma concentration) with cardiovascular health outcomes makes comparisons with prior findings difficult. Nevertheless, we consider that stratification by sex adds value to the conclusions, where the importance of sex-related differences is addressed.

The main strengths of the study are the precise carotenoid extraction and quantification in human plasma and the novel methodology for assessing the association with response variables taking into account that the values below the limits of quantification and detection are interval-censored data. These methods consider the whole interval of possible values instead of replacing it by a single imputed value such as the upper limit or the midpoint. As a consequence, the uncertainty of the estimated regression coefficients reflects the fact that the carotenoids below limits of quantification and detection are observations between zero and the limit of detection or between the limit of detection and the limit of quantification.

In summary, we crossectionally described associations between markers of cardiovascular health and plasma carotenoid concentrations in a sub-sample of participants from the PREDIMED-Plus study at baseline. Assesment of larger samples from both clinical trials and prospective cohorts is needed to support our findings and provide mechanistical insight in their molecular basis.

## Data availability statement

The datasets presented in this article are not readily available because there are restrictions on the availability of data for the PREDIMED-Plus trial, due to the signed consent agreements around data sharing, which only allow access to external researchers for studies following the project purposes. Requestors wishing to access the PREDIMED-Plus trial data used in this study can make a request to the PREDIMED-Plus trial Steering Committee chair: jordi.salas@urv.cat. The request will then be passed to members of the PREDIMED-Plus Steering Committee for deliberation. Requests to access the datasets should be directed to jordi.salas@urv.cat.

## Ethics statement

The studies involving human participants were reviewed and approved by Institutional Review Boards of the participating centers. The patients/participants provided their written informed consent to participate in this study.

## Author contributions

MM-M: conceptualization, formal analysis, investigation, methodology, software, visualization, and writing—original draft. ID-L: investigation and writing—review and editing. KL: software and writing—review and editing. AT-R: conceptualization and writing—review and editing. MZ, ER, ET, MFPC, RB, ET, LT-S, EG-G, JZ, MM, AG-R, RC, AG-P, JS-L, ZV-R, AA, EA, MG-R, VB, AM-R, OL, NB, and FPL: writing—review and editing. MMG, JS-S, DC, JM, AA-R, JW, JeV, DR, JL-M, RE, FT, JL, LS-M, AB-C, JT, VM-S, XP, MD-R, PM-M, JoV, CV, and LD: funding acquisition, resources, and writing—review and editing. MF: data curation, funding acquisition, and writing—review and editing. GGM: validation and writing—review and editing. RL-R: conceptualization, funding acquisition, project administration, supervision, validation, and writing—review and editing. All authors contributed to the article and approved the submitted version.

## Funding

This research was funded (AGL2016-75329-R and PID2020-114022RB-I0) and CIBEROBN from the Instituto de Salud Carlos III, ISCIII from the Ministerio de Ciencia, Innovación y Universidades, (AEI/FEDER 10.13039/501100011033, UE), Generalitat de Catalunya (GC) (2017SGR196, 2017SGR622). The PREDIMED-Plus trial was supported by the official Spanish Institutions for funding scientific biomedical research, CIBER Fisiopatología de la Obesidad y Nutrición (CIBEROBN) and Instituto de Salud Carlos III (ISCIII), through the Fondo de Investigación para la Salud (FIS), which is co-funded by the European Regional Development Fund (six coordinated FIS projects lead by JS-S and JoV, including the following projects: PI13/00673, PI13/00492, PI13/00272, PI13/01123, PI13/00462, PI13/00233, PI13/02184, PI13/00728, PI13/01090, PI13/01056, PI14/01722, PI14/00636, PI14/00618, PI14/00696, PI14/01206, PI14/01919, PI14/00853, PI14/01374, PI14/00972, PI14/00728, PI14/01471, PI16/00473, PI16/00662, PI16/01873, PI16/01094, PI16/00501, PI16/00533, PI16/00381, PI16/00366, PI16/01522, PI16/01120, PI17/00764, PI17/01183, PI17/00855, PI17/01347, PI17/00525, PI17/01827, PI17/00532, PI17/00215, PI17/01441, PI17/00508, PI17/01732, PI17/00926, PI19/00957, PI19/00386, PI19/00309, PI19/01032, PI19/00576, PI19/00017, PI19/01226, PI19/00781, PI19/01560, PI19/01332, PI20/01802, PI20/00138, PI20/01532, PI20/00456, PI20/00339, PI20/00557, PI20/00886, PI20/01158); the Especial Action Project entitled: Implementación y evaluación de una intervención intensiva sobre la actividad física Cohorte PREDIMED-Plus grant to JS-S; the European Research Council (Advanced Research Grant 2014–2019; agreement #340918) granted to MM-G.; the Recercaixa (number 2013ACUP00194) grant to JS-S; grants from the Consejería de Salud de la Junta de Andalucía (PI0458/2013, PS0358/2016, PI0137/2018); the PROMETEO/2017/017 grant from the Generalitat Valenciana; the SEMERGEN grant; None of the funding sources took part in the design, collection, analysis, interpretation of the data, or writing the report, or in the decision to submit the manuscript for publication. JS-S is partially supported by ICREA under the ICREA Academia programme. AT-R is a Serra Húnter fellow. MM-M is supported by the FPU17/00513 grant.

## Conflict of interest

JS-S reported receiving research support from the Instituto de Salud Carlos III, Ministerio de Educación y Ciencia, Departament de Salut Pública de la Generalitat de Catalunya, the European Commission, the USA National Institutes of Health; receiving consulting fees or travel expenses from Eroski Foundation, Instituto Danone, Mundipharma, receiving non-financial support from Hojiblanca, Patrimonio Comunal Olivarero, the Almond Board of California, Pistachio Growers and Borges S.A.; serving on the board of and receiving grant support through his institution from the International Nut and Dried Foundation and the Eroski Foundation; and personal fees from Instituto Danone; Serving in the Scientific Board of Danone Institute International. DC reported receiving grants from Instituto de Salud Carlos III. RE reported receiving grants from Instituto de Salud Carlos III, Fundación Dieta Mediterránea and Cerveza y Salud and olive oil for the trial from Fundación Patrimonio Comunal Olivarero and personal fees from Brewers of Europe, Fundación Cerveza y Salud, Interprofesional del Aceite de Oliva, Instituto Cervantes in Albuquerque, Milano and Tokyo, Pernod Ricard, Fundación Dieta Mediterránea (Spain), Wine and Culinary International Forum and Lilly Laboratories; non-financial support from Sociedad Española de Nutrición and Fundación Bosch y Gimpera; and grants from Uriach Laboratories. ER reports grants, personal fees, non-financial support, and other from California Walnut Commission, during the conduct of the study; grants, personal fees, non-financial support, and other from Alexion; personal fees and other from Amarin, outside the submitted work. RL-R reports personal fees from Cerveceros de España, personal fees and other from Adventia, other from Ecoveritas, S.A., outside the submitted work. The remaining authors declare that the research was conducted in the absence of any commercial or financial relationships that could be construed as a potential conflict of interest.

## Publisher's note

All claims expressed in this article are solely those of the authors and do not necessarily represent those of their affiliated organizations, or those of the publisher, the editors and the reviewers. Any product that may be evaluated in this article, or claim that may be made by its manufacturer, is not guaranteed or endorsed by the publisher.
